# Inverse Modeling for Artifact Removal in Photonic Data: A Computational Physics and Transfer Learning-Based Approach

**DOI:** 10.1021/acs.jcim.5c02055

**Published:** 2025-10-28

**Authors:** Ravi Teja Vulchi, Volodymyr Morgunov, Julian Hniopek, Oleg Ryabchykov, Thomas Bocklitz

**Affiliations:** 1 Institute of Physical Chemistry (IPC) and Abbe Center of Photonics (ACP), 9378 Friedrich Schiller University Jena, Member of the Leibniz Centre for Photonics in Infection Research (LPI), Helmholtzweg 4, Jena 07743, Germany; 2 Leibniz Institute of Photonic Technology, Member of Leibniz Health Technologies, Member of the Leibniz Centre for Photonics in Infection Research (LPI), Albert‑Einstein‑Strasse 9, Jena 07745, Germany

## Abstract

Etaloning artifacts introduce notable distortions in spectroscopic data, complicating downstream analysis and interpretation. We present an inverse modeling framework that integrates computational physics with deep learning to address this challenge. Our approach employs a two-phase transfer learning strategy: pretraining on over 30,000 simulated spectra generated using the transfer matrix method and fine-tuning on real experimental data. This extensive simulated data set enhances the model’s ability to generalize across different sensor designs, significantly improving robustness and accuracy. Rigorous cross-validation across multiple devices demonstrates that the transfer learning approach reduces etaloning-induced distortions by up to 70%, ensuring substantial spectral accuracy and interpretability improvements. This study sets a new standard for achieving reliable spectral data by combining correction procedures with physics simulations.

## Graphical abstract

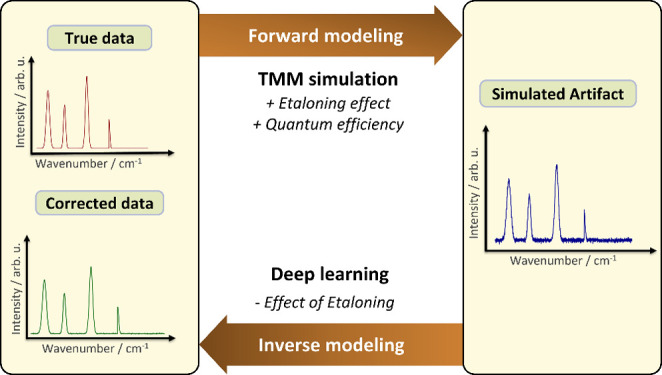

## 1 Introduction

Precise spectral measurements are essential across various scientific and technological fields, including biomedical diagnostics, remote sensing, next-generation imaging systems, and fundamental materials research. Among various spectroscopic techniques, Raman spectroscopy stands out for its ability to provide a molecular fingerprint, placing it at the forefront of medical diagnostics,^1^ pharmaceutical science,^2^ materials science,^3^ environmental monitoring,^4^ and forensic analysis.^5^ Despite these advancements, consistently high-quality data remains difficult to obtain due to artifacts.^6^

Etaloning is a prominent artifact caused by light reflecting multiple times within the layered structure of charge-coupled device (CCD) detectors. It produces interference fringes that distort the spectral data. These fringes, particularly noticeable in back-illuminated CCDs at longer wavelengths, can shift signal intensities by 15% due to the detector’s low quantum efficiency (QE).^7^ As demonstrated in Figure 1, artifacts such as the shot noise and periodic fringes, potentially arising from experimental or detector-related effects, obscure molecular features, complicating direct spectral interpretation. Developing and validating correction procedures requires a standard reference signal (ground truth). In this context, methods capable of reconstructing the underlying signals without relying on idealized benchmarks are needed.^8^

**Figure 1:**
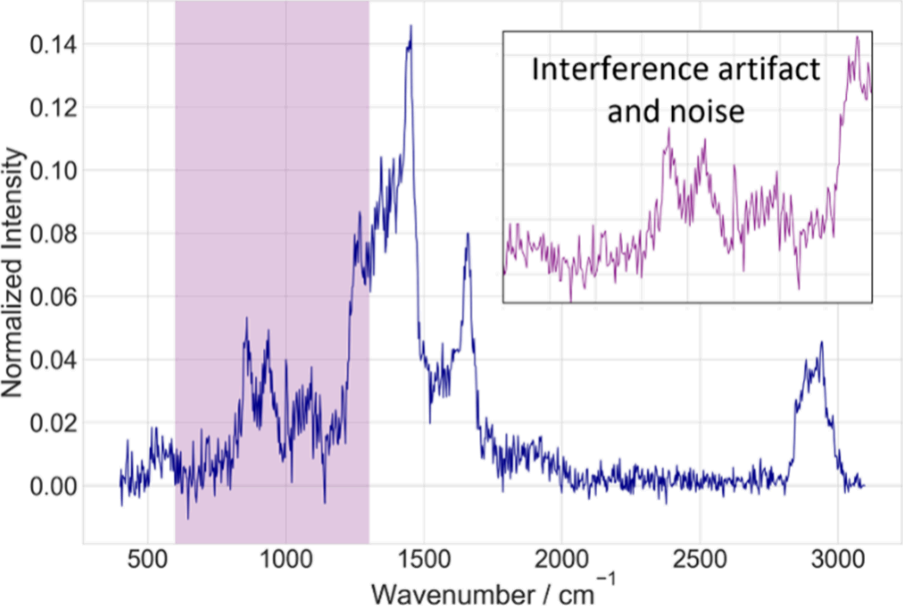
Preprocessed Raman spectrum of a biological sample after baseline correction and cosmic spike removal, illustrating the persistent artifacts in the spectral data. While key vibrational peaks are visible, the spectrum remains compromised by periodic fringes and noise, which may stem from detector or experimental artifacts necessitating correction methods, such as inverse modeling, to reconstruct artifact-free spectra.

In scientific data analysis, inverse modeling offers a more controlled approach to accurate parameter reconstruction under nonideal conditions by integrating well-defined physical models with mathematical frameworks. Given a forward model *y* = *f*(*x*), often nonlinear or only partially known, which maps system parameters *x* to measurable outputs *y*, inverse modeling aims to solve *x* = *f* ^–1^(*y*).^9^

In this context, forward modeling with the transfer matrix method (TMM) generates synthetic data by simulating light interactions within multilayered optical systems, thus incorporating artifacts such as etaloning into the data set. Compared to general Maxwell equation solvers such as finite-difference time-domain, TMM offers significant advantages in modeling 1D-layered structures due to its high computational efficiency and ability to directly compute frequency-domain responses.^10^ This efficiency is further amplified by optimized Python implementations, such as tmm_fast,^11^ making TMM a highly practical choice for generating the large, physically realistic synthetic data sets required to train correction models.

Neural networks have found significant applications in inverse modeling, particularly for solving complex, nonlinear problems where analytical solutions are impractical.^12^ Unlike analytical models, which require explicit formulas and often struggle with high-dimensional, nonlinear design spaces, deep learning (DL) models can learn the inverse mapping directly from the data, often surpassing the performance of classical inversion methods. For Raman spectral correction specifically, the U-Net architecture has proven particularly effective.^13^ Its success is due to its special encoder-decoder design with skip links, a configuration that allows the capture of the global spectral context and intricate local features. By generating synthetic data sets through TMM-based forward modeling, we establish labeled ground truths for training the U-Net architecture and then apply transfer learning (TL) to generalize effectively. Furthermore, acquiring the large-scale-labeled data sets of real spectra required for direct training is frequently impractical. Transfer learning addresses these limitations by allowing the model to leverage knowledge gained from related data.^14^ This involves pretraining the U-Net model on extensive synthetic data generated via the TMM, enabling it to learn the fundamental physics-based artifact removal mapping. Subsequently, the model is fine-tuned by using a limited set of real experimental spectra. This combination of TMM with deep learning-based TL represents a novel methodology for precise etaloning correction.

### 1.1 Scope of This Study

The present study focuses on introducing a unified framework that integrates inverse modeling with computational physics and DL to address the challenge of etaloning artifacts in Raman spectra. Building on a physics-based simulation pipeline, we designed a transfer learning strategy in which the U-Net model learns to identify and correct fringe patterns by being trained on spectra containing well-defined interference effects. These fringes serve as structured signals that guide learning within the network, where shallow layers focus on localized interference features and deeper layers capture the broader spectral context needed for precise correction.

To ensure the model’s generalization across detector types and acquisition settings, we implement a leave-one-CCD-out validation strategy, which systematically evaluates performance on unseen CCD designs and mitigates potential biases in training data composition. Rather than comparing this with general artifact removal techniques not designed for this specific task, we demonstrate the unique benefit of coupling physics-driven simulation with a DL model refined through transfer learning. The full two-stage procedure, including the forward simulation of etaloning effects and the inverse modeling strategy using transfer learning, is illustrated in Figure 2.

**Figure 2:**
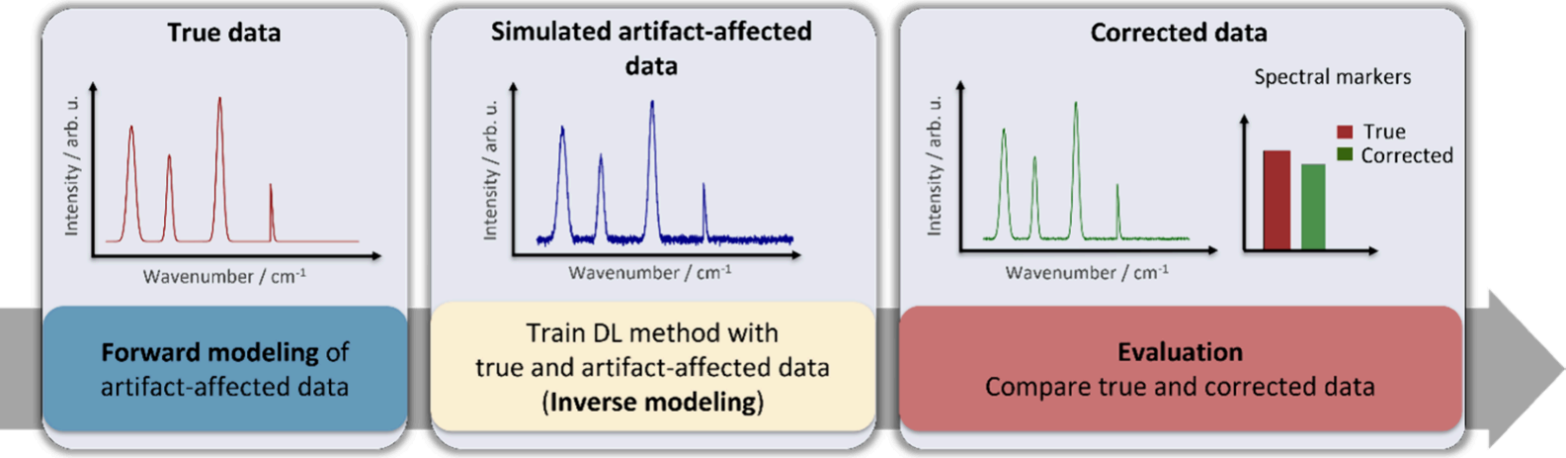
Schematic representation of the two-stage workflow for correcting etaloning artifacts in Raman spectroscopy. In the forward modeling phase, we simulate etaloning affected data for each data set type by employing distinct steps and parameters tailored to their specific experimental conditions. Steps include TMM-based processes, introducing etaloning fringes and QE variations, adding fluorescence baselines, and applying dark current noise removal (see Section 2.3.3. for details). During the inverse modeling phase, a U-Net model is pretrained on synthetic data sets with known ground truths and then fine-tuned using a limited set of real experimental spectra. As a substage of inverse modeling, the final step evaluates the model’s correction performance by comparing the reconstructed output with the known true spectra through spectral markers and metrics.

For completeness, we also evaluated a simple, nonphysics-based baseline algorithm. Savitzky–Golay (SG) smoothing was applied to the Raman spectra with etaloning to produce a corrected spectrum. SG is a fixed window, low-pass, polynomial filter that requires tuning for each spectrum and still fails to separate interference from the genuine Raman structure. On representative spectra, small windows leave the etalon ripple clearly visible, medium windows reduce the ripple but blunt narrow bands and alter band ratios, and large windows oversmooth the signal, removing fine vibrational detail. Since etaloning is structured and wavenumber-dependent, a single fixed window cannot eliminate it without degrading the spectrum. See the Supporting Information Section “Savitzky–Golay Smoothing as a Baseline for Etaloning Removal” and Figure S1 for illustrative examples.

## 2 Materials and Methodology

### 2.1 Simulated Data Set for Pretraining

A simulated spectral data set was generated to pretrain the DL model, embedding a physics-informed understanding of spectral behavior. Spectra were generated using the GFN2-xTB semiempirical quantum mechanical method, which offers a favorable balance between computational speed and accuracy by approximating correlation integrals through a tight-binding approach with empirical corrections. This method enables the efficient calculation of vibrational spectra for multiple small molecules.^15^

We simulated over 30,000 spectra for molecules sourced from the small-molecule set of the World Wide Protein Data Bank (wwPDB), encompassing a chemically diverse range of biologically relevant compounds typically containing up to 100 atoms. For each molecule, geometry optimization followed by vibrational frequency calculations was performed to obtain normal modes (see the Supporting Information, “Spectral Data Simulation,” for full computational details). The simulated spectra encompass various molecular structures, vibrational modes, and spectral conditions, creating a comprehensive training data set that reflects realistic complexities.

The spectra were generated by quantum mechanical calculations and broadening effects were modelled. In order to generate Raman spectra that are consistent with actual experimental results, the discrete vibrational transitions were broadened by using Lorentzian line profiles. A full width at half-maximum (fwhm) was assigned to each peak, with the fwhm being randomly sampled in the range of 6–80 cm^–^^1^ (γ = 3–40 cm^–^^1^).

Additionally, spectra were altered by introducing noise, peak shifts, and silent regions to enhance the variability of the data. This variability mimics real-world conditions, ensuring that the model learns to handle diverse spectral artifacts. Figure 3a depicts the mean spectrum of the simulated spectra with a shaded region representing the standard deviation. The spectral range spans 0–3700 cm^–^^1^, capturing both fundamental and overtone vibrational modes. Negative values in the shaded area, resulting from statistical processing, lack physical meaning, as Raman intensities are inherently non-negative.

**Figure 3:**
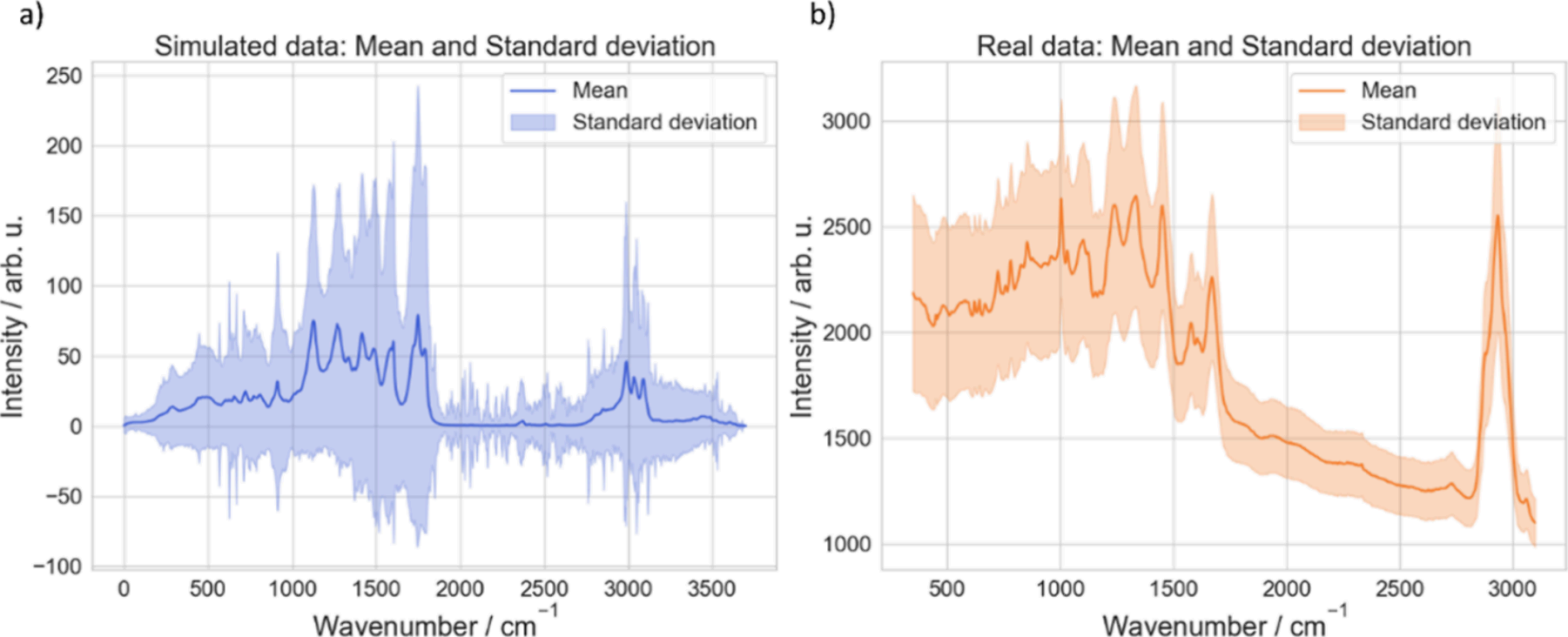
Representation of the data sets utilized for training and fine-tuning in the etaloning artifact correction workflow. (a) Mean Raman spectrum of the simulated data set (blue line) derived from over 30,000 synthetic spectra. The shaded region represents one standard deviation above and below the mean, highlighting the spectral variability. The spectral range of 0–3700 cm^–^^1^ includes fundamental and overtone bands. (b) Mean Raman spectrum of *Escherichia coli* from batch 1 (orange line) measured using a 532 nm excitation laser with a spectral resolution of 8 cm^–^^1^. The shaded region reflects biological and experimental variability within the batch.

### 2.2 Real Experimental Data Set for Fine-Tuning

This study utilized a real experimental Raman spectral data set to fine-tune the DL model for addressing etaloning artifacts. The data set provides a critical benchmark for evaluating the model’s generalization under real-world experimental conditions. Derived from a bacterial classification study, the data set includes six bacterial species (*Escherischia coli*, *Klebsiella terrigena*, *Pseudomonas stutzeri*, *Listeria innocua*, *Staphylococcus warneri*, and *Staphylococcus cohnii*), cultivated in nine biological replicates (batches). 2652 Raman spectra were recorded under controlled conditions using a 532 nm excitation laser with a spectral resolution of approximately 8 cm^–^^1^. While etaloning effects are absent at this wavelength, the data set captures significant spectral variability due to biological and experimental factors, making it an invaluable resource for model validation.

Preprocessing was performed to ensure data consistency while preserving the baseline information. This included cosmic spike removal and wavenumber calibration to correct for spectral misalignments. The data set’s hierarchical organization supports advanced validation protocols, such as Leave-One-Batch-Out Cross-Validation, which ensures independence between training and testing data and provides reliable estimates of model generalization.^16^ Figure 3b presents the mean Raman spectrum of *E. coli* from batch 1, illustrating characteristic spectral features overlaid with the standard deviation as the shaded region. Further details about the data set can be found in ref 17. This visualization highlights the spectral consistency within the batch and the variability arising from the inherent biological and experimental differences. Such variability emphasizes the importance of robust preprocessing and model fine-tuning to generalize across diverse spectral conditions.

### 2.3 Simulating Artifacts: Forward Modeling of Absorption and Etaloning Effects

In this section, we simulate absorption and etaloning effects in Raman spectra by forward modeling the light propagation through multilayer structures using the TMM, an approach efficiently implemented via the tmm_fast package (see the Supporting Information, “Software Packages and Computational Tools”) that leverages GPU acceleration, Autograd, and vectorized operations to replicate the interference patterns characteristic of back-illuminated CCD sensors. TMM propagates fields sequentially through matrix operations across each layer, leading to computational costs that scale linearly with the number of layers (see the Supporting Information, “Transfer Matrix Method as the Forward Modeling Framework”).

#### 2.3.1 Etaloning in Multilayer Structures and CCD Detectors

Due to their high sensitivity and accuracy, CCDs have become the standard for detecting weak inelastic scattering signals. These detectors are critical for spectroscopic applications, enabling the characterization of molecular vibrational modes and material properties under varying conditions. Back-illuminated CCDs, in particular, offer enhanced QE in the near-infrared (NIR) region, making them suitable for applications requiring deeper photon penetration. However, their design introduces etaloning, an interference artifact that distorts spectra, especially in the presence of weak Raman signals or fluorescence.

Figure 4 provides a detailed depiction of the multilayer structure and the physical mechanism underlying etaloning. Etaloning arises from internal reflections within the multilayer structure of back-illuminated CCDs, where light reflects at interfaces between layers with differing refractive indices. These reflections create interference patterns, alternating bright and dark fringes that depend on the wavelength of the incident light and the optical path differences. Long-wavelength lasers, such as 785 nm, are particularly sensitive to this effect due to their more profound penetration into the active layers of the CCD. Fringing, an effect of etaloning, becomes especially apparent in samples with significant fluorescence.^18^ Fluorescence often introduces a baseline in the Raman spectrum, obscuring weaker signals. Even after baseline correction, residual shifts combined with weak Raman intensities can make fringing artifacts more prominent.

**Figure 4:**
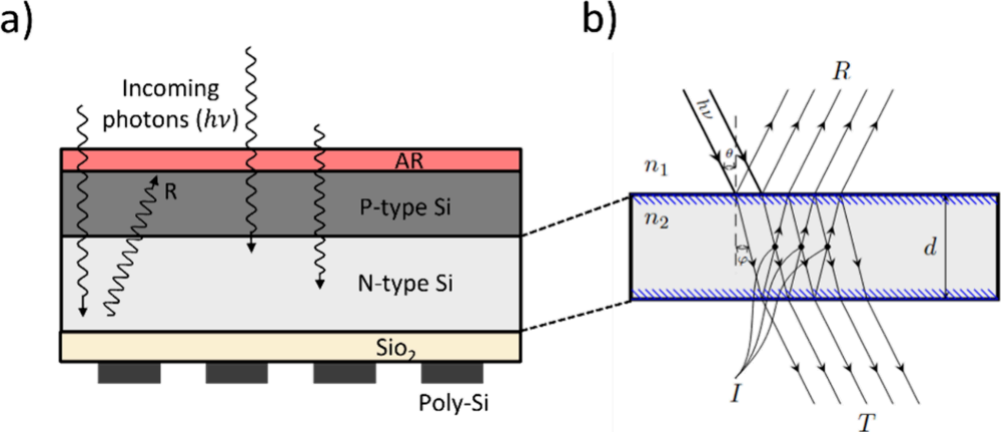
Illustrations of a back-illuminated CCD structure and the physics of the etaloning effect. (a) Cross-sectional schematic of a back-illuminated CCD, showing the key layers: The antireflective (AR) coating minimizes surface reflections, the p-type silicon serves as the light absorption region, the n-type silicon forms the depletion region for charge separation, and the SiO_2_ layer provides structural support. Internal reflections within these layers lead to interference patterns. (b) Physics of etaloning in the depletion region. The incident light (*h*ν) at angle θ is partially reflected (*R*) and transmitted (*T*) at interfaces between materials with refractive indices of *n*_1_ and *n*_2_. Constructive interference occurs when the phase difference between reflected waves corresponds to an integer multiple of 2π, amplifying the light intensity. Destructive interference occurs when the phase difference is an odd multiple of π, reducing the light intensity. These interference effects result in alternating bright and dark fringes, which characterize the etaloning artifact.

A comparison of front-illuminated and back-illuminated CCDs highlights the severity of this effect.^19^ Back-illuminated CCDs, while offering enhanced QE, are more prone to etaloning due to their structural design. For example, spectra from a 10 euro note acquired with a back-illuminated CCD reveal pronounced fringing even after baseline correction. In contrast, front-illuminated CCDs show minimal fringing by reducing the number of internal reflections. Figures 6 and 7 from ref 19 illustrate the impact of fringing on corrected spectra and spectral accuracy, underscoring the importance of selecting the appropriate CCD architecture for fluorescence samples.

#### 2.3.2 Modeling the Propagation of Light Using TMM

TMM is particularly effective for modeling electromagnetic wave propagation in layered media. When studying wave propagation in a multilayer structure, we express the electric field *E*(*z*) within a single layer as a superposition of forward- and backward-propagating waves, as shown in eq 1:

$$E(z)=E_{f}e^{ik_{f}z}+E_{b}e^{-ik_{b}z}$$ 1

Here, *E*_f_ and *E*_b_ represent the amplitudes of the forward- and backward-propagating waves, respectively, while *k*_f_ and *k*_b_ are the wave vectors. We take the axial wavevector to be $k_{n}=\frac{2\pi }{\lambda }n\cos\theta$ (for absorbing media, use *ñ* = *n* + *i* *k*_*n*_), and in a homogeneous layer set, *k*_f_= *k*_b_ = *k*_*n*_, at normal incidence (θ = 0), this reduces to $k_{n}=\frac{2\pi n}{\lambda }$ , where *n* denotes the refractive index of the medium and λ represents the wavelength of the incident light. This equationcaptures the phase changes experienced by the electromagnetic wave as it traverses a given layer.

At the interface between two layers with refractive indices *n*_1_ and *n*_2_, we use the Fresnel equations to calculate the reflection (*r*_s_) and transmission (*t*_s_) coefficients for s-polarized light (see eqs 2 and 3). Only the s-polarized expressions are given here; the corresponding p-polarized forms are analogous.

$$r_{s}=\frac{n_{1}\cos\theta_{1}-n_{2}\cos\theta_{2}}{n_{1}\cos\theta_{1}+n_{2}\cos\theta_{2}}$$ 2

$$t_{s}=\frac{2n_{1}\cos\theta_{1}}{n_{1}\cos\theta_{1}+n_{2}\cos\theta_{2}}$$ 3

In these equations, θ_1_ and θ_2_ represent the angles of incidence and refraction, respectively, and they satisfy Snell’s law (*n*_1_ sin θ_1_ = *n*_2_ sin θ_2_). These coefficients describe the interaction of light at a single boundary, providing a foundation for complex multilayer models. We define the transfer matrix ***M***_***n***_ (see eq 4) to describe how waves propagate through a single layer and across the interface to layer *n* + 1. Physically (see eq 4), *M*_*n*_ combines two effects: propagation through layer *n,* which adds a phase shift of δ_*n*_, and the interface between layers *n* and *n* + 1, characterized by the Fresnel coefficients *r*_*n*,*n*+1_ and *t*_*n*,*n*+1_. In other words, *M*_*n*_ maps the forward- and backward-propagating field amplitudes from layer *n* to layer *n* + 1.

$$M_{n}=\left(\begin{array}{cc} e^{-i\delta_{n}} & 0\\ 0 & e^{i\delta_{n}} \end{array}\right)\left(\begin{array}{cc} 1 & r_{n,n+1}\\ r_{n,n+1} & 1 \end{array}\right)\frac{1}{t_{n,n+1}}$$ 4

where δ_*n*_ = *k*_*n*_ *d*_*n*_ represents the phase shift introduced by a layer of thickness *d*_*n*_. *r*_*n*,*n*+1_ and *t*_*n*,*n*+1_ are the reflection and transmission coefficients at the boundary between layers *n* and *n* + 1. This transfer matrix accounts for the phase changes within the layer and the effects of reflection and transmission at its interfaces. For a system composed of multiple layers, we calculate the overall transfer matrix by *M*, multiplying the transfer matrices of each layer (see eq 5). The product is ordered from the incident medium toward the substrate, so *M*_0_ acts first on the incident field.

$$M=M_{0}M_{1}\cdots M_{N-1}$$ 5

*M*_0_ *and M*_*N*–1_ encode the boundary conditions at the incident medium and the substrate. We impose unit incident amplitude and no backward-propagating wave in the substrate, leading to:

$$\left(\begin{array}{c} 1\\ r \end{array}\right)=M\left(\begin{array}{c} t\\ 0 \end{array}\right)$$ 6

This relation determines how the electric field amplitudes evolve through the multiple layersand enables computation of the overall reflection (*r*) and transmission (*t*) (eq 6).

From the elements of the total transfer matrix, we extract the reflection (*r*) and transmission (*t*) coefficients as follows:

$$r=\frac{M_{21}}{M_{11}}$$ 7

$$t=\frac{1}{M_{11}}$$ 8

Here, ***M***_*ij*_ denotes the element of the stack transfer matrix ***M*** in row *i*, column *j*, using the standard 1-based convention (so ***M***_11_ is the top-left and ***M***_21_ is the lower-left element). These coefficients quantify the proportions of light reflected and transmitted by the multilayer system. To evaluate the energy behavior of the system, we calculated the transmission (*T*) and reflection (*R*) coefficients (see eqs 9 and 10).

T=|t|2Re(ncosθ)Re(n0cosθ0) 9

$$R={\left|r\right|}^{2}$$ 10

A=1−R−T 11

where *n*_0_ and θ_0_ are the initial medium’s refractive index and incidence angle, respectively. These equations incorporate the change in impedance between the incident and transmitted media, ensuring that the energy conservation principles are obeyed. Finally, we calculate the absorbance (*A*) (see eq 11) as the difference between the incident power and the sum of reflected and transmitted power. Multiple internal reflections encoded by ***M*** produce interference fringes (etaloning) with approximate wavenumber spacing set by optical thickness and amplitude governed by the interface reflectivities.

Assumptions and limitations of the TMM used here (e.g., planar homogeneous layers, coherent illumination, and ideal interfaces) are summarized in the Supporting Information (“Transfer Matrix Method as the Forward Modeling Framework”). For more in-depth information on the theoretical background of TMM, refer to the chapter on thin films in micro photonics, studies on multilayer optical computations,^20^ and the analysis of Fresnel coefficients in thin films.^21^ Since the precise specifications of CCDs used in Raman spectroscopy are not open source, we based our simulations on astronomical CCD designs optimized for a broad spectral range (300–1100 nm). We collected available CCD parameters from referenced sources and categorized them into interpolation and extrapolation scenarios.^7,22^

In interpolation scenarios (Designs 6–8), we modified the silicon layer thickness within a range (14,000–18,000 nm) while keeping the material compositions and structures consistent with known CCD configurations. For example, Design 7 features variations in silicon thickness while retaining standard layers, such as ITO, SiO_2_, and Si_3_N_4_. These modifications simulate etaloning patterns within a familiar design space, allowing the model to predict interference patterns based on known configurations. In extrapolation scenarios (Designs 1–4), we introduced new materials and layer compositions, such as high-refractive-index layers such as TiO_2_ and ZrO_2_, and entirely new multilayer structures. These configurations extended beyond typical CCD parameters, testing the model’s ability to generalize to unfamiliar etaloning patterns. Table 1 summarizes the material properties of CCD designs, illustrating examples of interpolation (Design 7) and extrapolation (Design 4) scenarios. These designs exemplify the structural variations and material compositions employed in cross-validation to assess the model’s capacity to handle familiar and unfamiliar etaloning patterns. Remaining CCD designs and their material configurations are provided in Table S1 of the Supporting Information.

**Table 1:** Material Properties of CCD Designs that Illustrate Interpolation (Design 7) and Extrapolation (Design 4) Scenarios^a^

designs	number of layers	materials used	thickness of layers (in order)
design 4	8	TiO_2_, SiO_2_, ISDP, SiO_2_ (additional), Si_3_N_4_, polysilicon gates, additional SiO_2_, silicon substrate	57 nm, 132 nm, 32 nm, 50 nm, 50 nm, 300 nm, 2 μm, 200 μm
design 7	7	ITO, SiO_2_, ISDP, silicon bulk, SiO_2_, Si_3_N_4_, silicon substrate	80 nm, 55 nm, 10 nm, 16 μm, 80 nm, 240 nm, 500 nm

a These designs exemplify the structural variations and material compositions employed in cross-validation to assess the model’s capacity to manage familiar and unfamiliar etaloning patterns.

To illustrate the TMM’s application in modeling etaloning patterns, we compare simulated and experimental data sets for Design 7, shown in Figure 5. The simulations are conducted using a laser excitation wavelength of 785 nm, typical for Raman spectroscopy, but the fringes are notably larger because the CCD is optimized for the broader spectral range of 300–1100 nm for astronomical applications.

**Figure 5:**
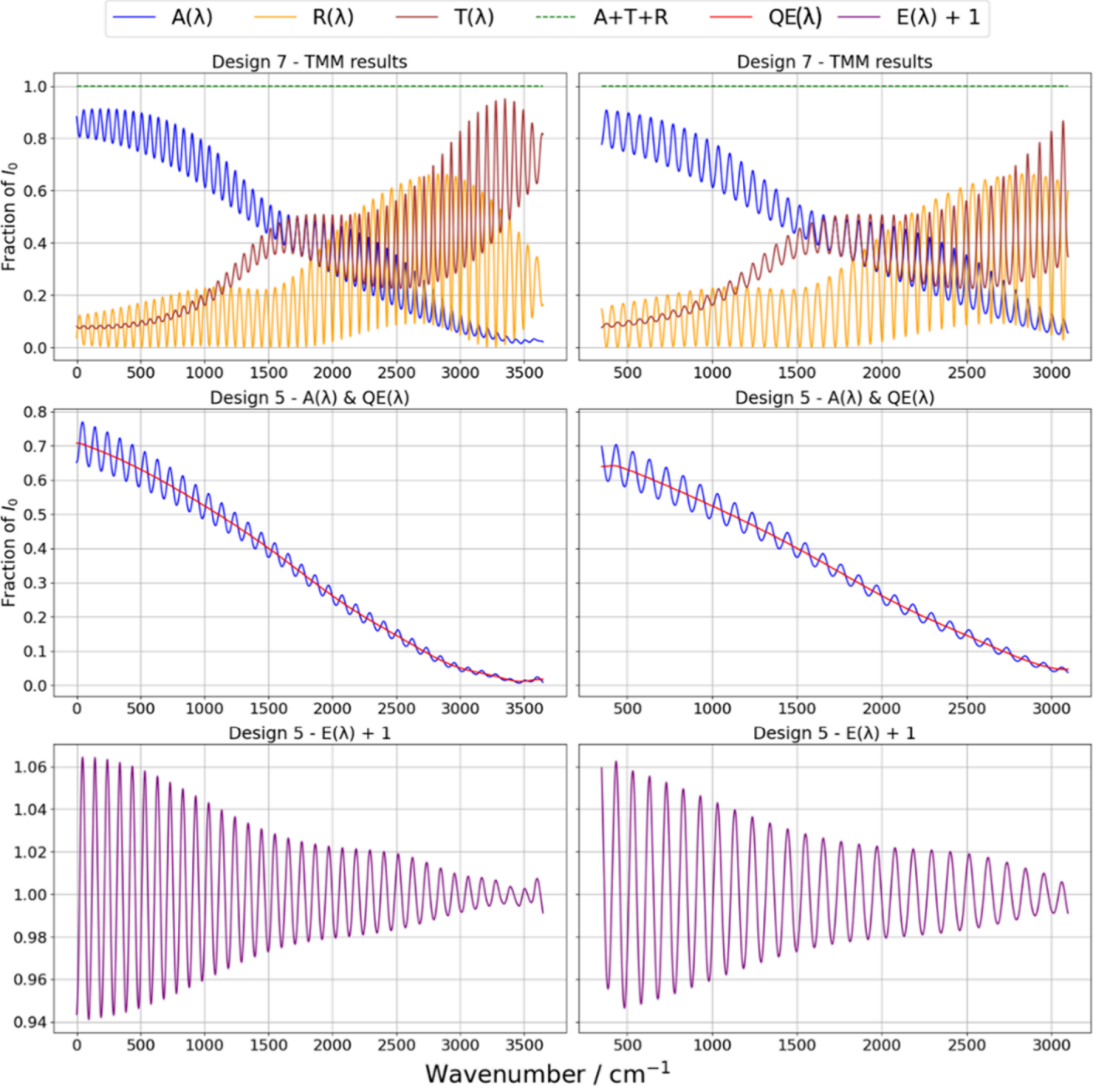
Results of TMM simulations for two CCD designs, illustrating the optical properties and etaloning effects across the spectral ranges of simulated and real experimental data sets. The top row of the figure displays *R*(λ), *A*(λ), and *T*(λ) for Design 7 as fractions of the incident intensity *I*_0_, with the sum *R* + *A* + *T* = 1 (green dashed line) confirming energy conservation. Simulated data (left) span the spectral range 785–1099 nm (000–3648 cm^–1^), while experimental data (right) cover 800–1037 nm (350–3098 cm^–1^). The central panels present the results for Design 5, and *A*(λ) and QE­(λ) are presented, where QE­(λ) is derived by applying a Gaussian filter to *A*(λ). The relative etaloning effect, defined as *E*(λ) = *A*(λ) – QE­(λ), quantifies interference-induced fringes by isolating the contribution of etaloning from the overall absorbance. The bottom row of the figure presents the *E*(λ) + 1 curves, which are plotted to shift the relative etaloning effect into a positive range, thereby enabling visualization of both the magnitude and periodicity of fringes. The presence of pronounced etaloning fringes is observed at lower wavenumbers, gradually decreasing at higher wavenumbers.

We adjusted the material properties from the interpolation scenarios to create Design 5 to reduce interference fringes. By introducing thinner layers for AR coatings, such as ITO, ZrO_2_, and SiO_2_, and reducing the thickness of the depletion layer (Si substrate), we suppressed etaloning-induced fringes in the higher wavenumber region while maintaining their prominence in the lower wavenumber range. These targeted adjustments allowed the resulting spectra to closely resemble realistic Raman data with controlled interference patterns, as seen in Figure 7.

#### 2.3.3 Forward Modeling of Synthetic Data Sets

We generated the simulated data set to pretrain DL models by replicating etaloning artifacts and fluorescence baselines observed in experimental Raman spectra. Starting with synthetic Raman spectra, we applied a fluorescence baseline to simulate the background. Next, we multiplied these spectra with absorbance coefficient *A*(λ), which integrates QE and *E*(λ). This operation introduced the interference fringes characteristic of CCD sensor responses, particularly at longer wavelengths. The resulting data set pairs spectra with etaloning effects and their corresponding ground truth spectra, allowing supervised learning in a controlled environment. Figure 6a outlines this workflow, while Figure 6b demonstrates how spectra from two samples, each with distinct fluorescence levels and intensities, were processed to illustrate the effect of etaloning. The simulated data set is optimized explicitly for pretraining DL models, enabling them to learn fundamental patterns and interference effects in a controlled setting.

**Figure 6:**
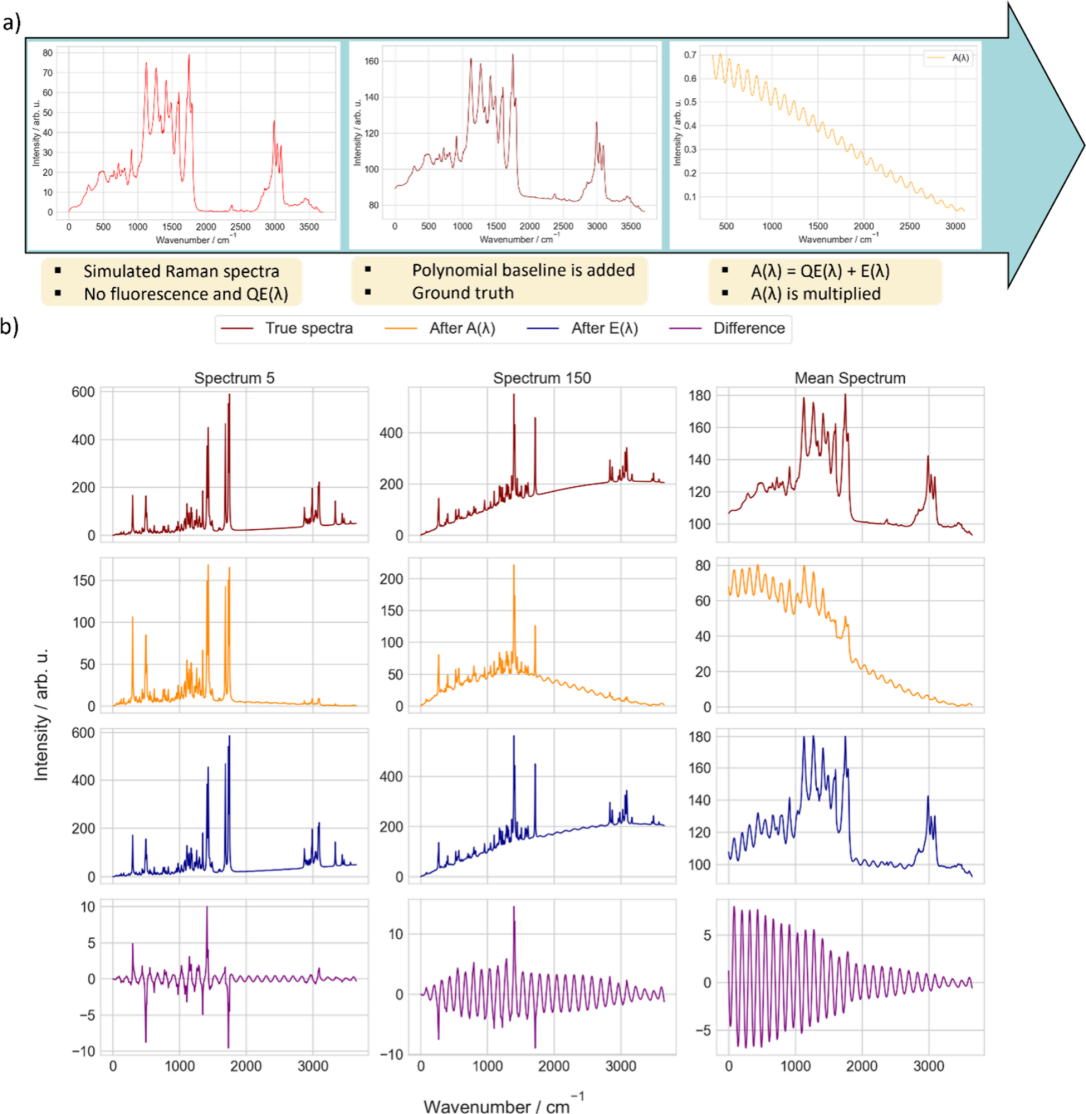
Workflow of forward modeling and example spectra from the simulated data set. (a) Key steps in forward modeling are illustrated for generating the simulated data set. Starting with simulated Raman spectra, a polynomial fluorescence baseline is added to mimic the experimental conditions. Etaloning artifacts are introduced by multiplying with *A*(λ), which incorporates both QE­(λ) and *E*(λ). This process produces interference fringes characteristic of CCD sensors, creating realistic training data for artifact correction. (b) Example spectra from the simulated data set for two samples (Spectrum 5 and Spectrum 150) with distinct fluorescence levels and the mean spectrum of the data set. The first row shows the true spectra without artifacts. The second row presents spectra modified with *A*(λ), which simulate the combined effects of QE­(λ) and *E*(λ) and serve as input to the DL model. The third row shows spectra with only *E*(λ), characterized by interference fringes. The final row highlights the differences between the true spectra and the spectra exhibiting etaloning artifacts, which are employed as a metric to assess the efficacy of the DL model. This data set is optimized for pretraining DL models to correct spectral artifacts.

We prepared the experimental data set using Raman measurements of six bacterial species across multiple biological replicates. These spectra inherently included fluorescence baselines and QE variations. we subtracted approximately 600 arbitrary units from the spectra to handle the dark current of the sensor. This preprocessing step mitigated the impact of sensor-related noise while preserving the spectral features. To isolate the etaloning patterns, we applied *E*(λ) = ((*A*(λ) – QE­(λ)) + 1), removing QE contributions and highlighting interference effects. Figure 7a also illustrates this preparation process, and Figure 7b provides examples of experimental spectra processed similarly to the simulated data set, including raw spectra, normalized spectra, and residual differences. The data set highlights two distinct samples with different fluorescence levels and intensities and a mean spectrum to represent the data set. This experimental data set is critical for fine-tuning models pretrained on simulated data. The fine-tuning process enhances the model performance by exposing it to the variability and complexity of real-world spectra, ensuring robustness and generalization.

**Figure 7:**
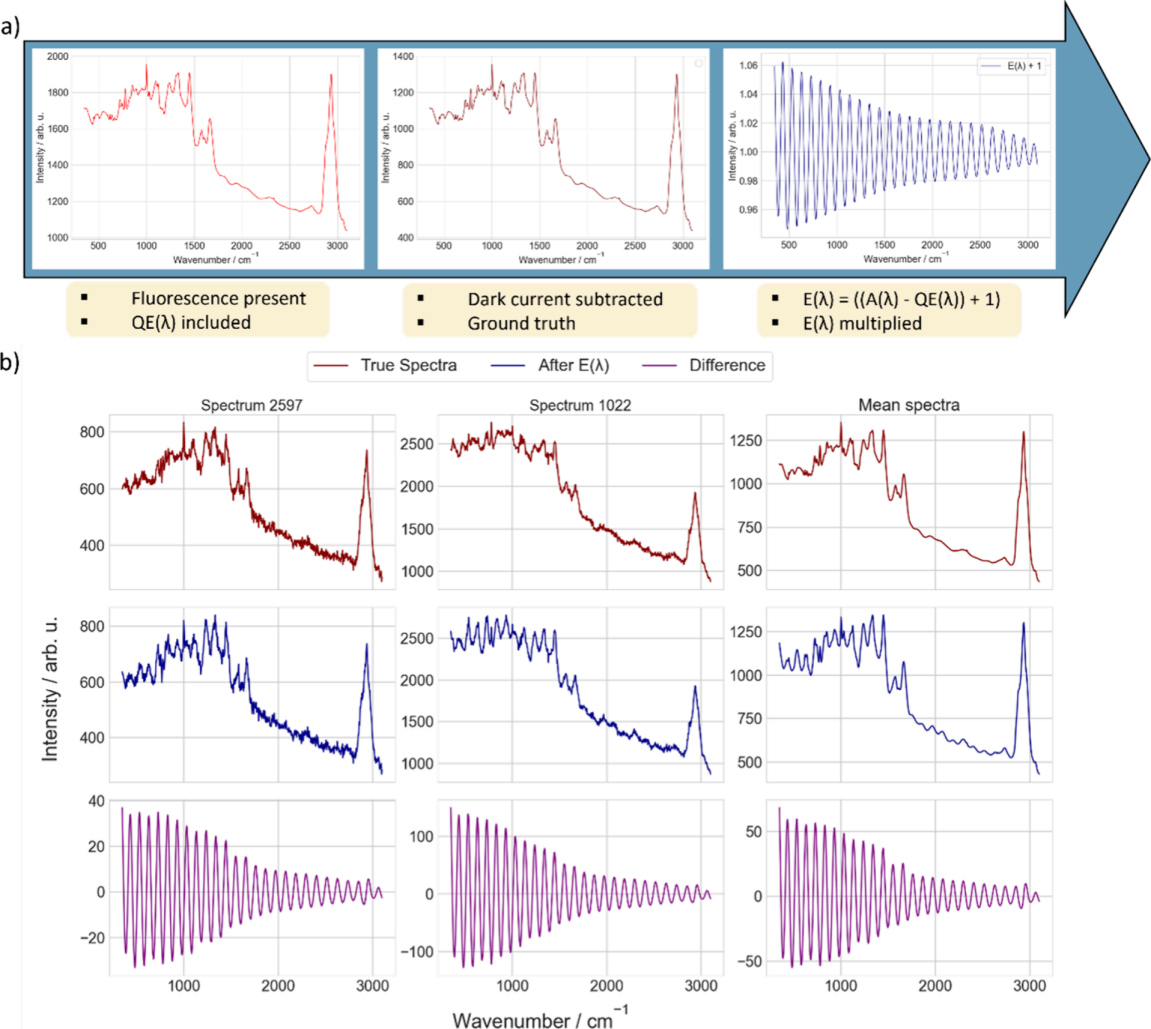
Workflow of forward modeling and example spectra from the real experimental data set. (a) Key steps in preparing the experimental data set are outlined. Experimental spectra inherently include fluorescence baselines and variations in the QE. A dark current of 600 arbitrary units was subtracted , which was estimated as a fixed offset. To isolate etaloning patterns, the spectra were multiplied using *E*(λ) = ((*A*(λ) – QE­(λ)) + 1), effectively removing contributions from the QE while preserving interference-induced fringes. This data set is used for fine-tuning (phase 2) DL models, ensuring they generalize effectively to real experimental conditions. (b) Example spectra from the experimental data set for two samples (Spectrum 2597 and Spectrum 1022) with distinct fluorescence levels and the mean spectra of the data set. The first row shows the experimental spectra, which include fluorescence baselines, QE variations, and etaloning patterns. The second row displays spectra processed using *E*(λ), which isolate the etaloning component while removing QE contributions; these spectra are sent as input to the DL model. The final row highlights the differences after applying *E*(λ), which serves as one of the metrics for evaluating the model’s performance in artifact correction. This workflow ensures that the DL model effectively learns to handle varying fluorescence levels and interference patterns, enabling robust artifact correction under real experimental conditions.

### 2.4 Inverse Modeling via Deep Learning

The U-Net architecture, initially developed for biomedical image segmentation, has been specifically adapted for one-dimensional (1D) spectral data to address the etaloning correction in Raman data. Key modifications include using 1D convolutional layers to process spectral input, tailored batch normalization for stabilizing feature distributions in data, and optimized dropout layers to prevent overfitting in smaller data sets. Additionally, the architecture incorporates domain-specific skip connections that preserve high-resolution spectral details critical for Raman signal integrity, ensuring accurate reconstruction, even after interference patterns are removed. This U-Net’s encoder-decoder structure enables simultaneous modeling of localized spectral features and broader interference patterns, making it well-suited for artifact correction in data-constrained regimes typical of spectroscopy applications. Concretely, we use five-level blocks (encoder channels 32–64–128–256–512 with a 1024-channel bottleneck), 2× down/upsampling per level, a transposed-convolution decoder with skip connections, and a 1 × 1 output convolution followed by softplus. All convolutions use a fixed odd-length kernel chosen to span several sampling intervals, large enough to capture long-period etaloning yet small enough to preserve narrow peaks.

TL, a well-established technique in DL, is employed here to bridge the gap between the synthetic and experimental data. By leveraging pretraining on simulated spectra, the U-Net learns generalized patterns of *A*(λ) and *E*(λ) artifacts in a controlled environment. This knowledge is transferred to the fine-tuning phase, where the model adapts to real experimental spectra, accommodating variabilities such as artifacts and sample-specific features. This approach significantly reduces the dependence on extensive experimental data sets, enhances model generalization, and ensures fine artifact removal even in challenging real-world conditions.

As illustrated in Figure 8, the TL workflow consists of two phases. The U-Net is trained on synthetic spectra featuring absorbance artifacts during pretraining, allowing the model to capture fundamental interference patterns. The learned weights are then fine-tuned on real spectra including *A*(λ) and *E*(λ) artifacts. The encoder path, with its five sequential blocks, captures spectral features, while the decoder path utilizes transposed convolutions and skip connections to reconstruct artifact-free spectra. This dual-phase framework removes etaloning artifacts and achieves high-fidelity spectral reconstruction as spectral metrics validate.

**Figure 8:**
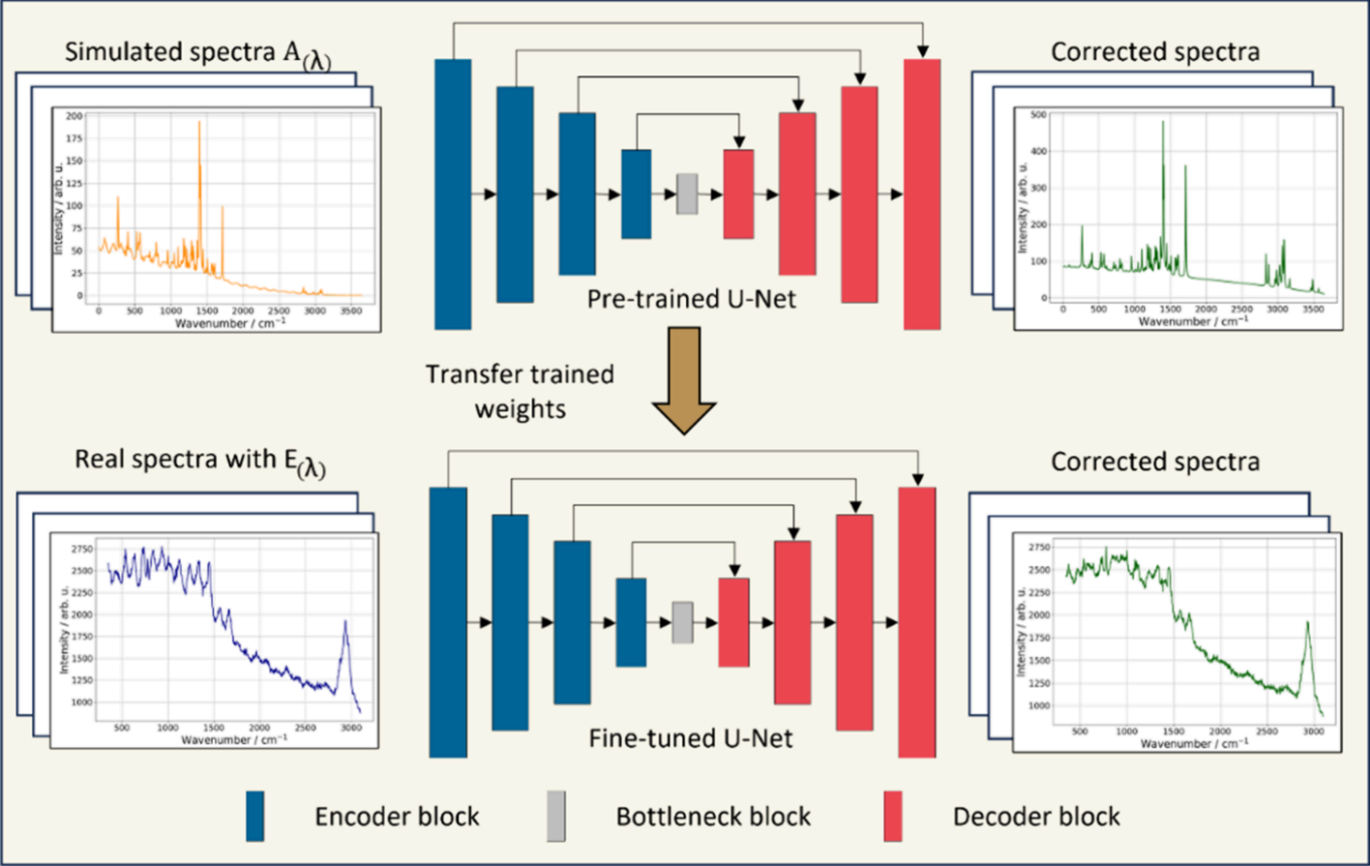
U-Net for the etaloning correction with two-phase transfer learning (TL). The model is a five-level 1D encoder-decoder with 2× down/upsampling, skip connections, and a bottleneck; the decoder uses transposed-convolution upsampling (see Table 2 for block details). Phase I (pretraining): learn *A*(λ)/interference patterns from simulated spectra. Phase II (fine-tuning): adapt the pretrained weights to experimental spectra containing *A*(λ) and *E*(λ).

#### 2.4.1 Hyperparameter Optimization

Finding the right hyperparameters is a well-known challenge in DL, as it involves striking a delicate balance. For instance, the learning rate has to be just right to ensure stable training, while the dropout rate needs to be high enough to prevent overfitting without affecting the model’s ability to learn. To navigate this complex trade-off effectively, we turned to a systematic, automated search. We optimized our DL model’s hyperparameters using Optuna, which employs an adaptive two-step process. First, it performs random sampling with 10 initial trials (n_startup_trials = 10) to broadly explore the search space. After this, Optuna switches to the Tree-Structured Parzen Estimator (TPE), a Bayesian optimization method that uses Gaussian mixture models to model promising and less promising hyperparameter values. TPE selects values by maximizing the ratio *l*(*x*)/*g*(*x*), effectively balancing exploration of diverse possibilities and the exploitation of promising regions. Optuna’s adaptive exploration, starting with the random trials and followed by TPE’s refined search, enabled us to determine an optimal combination of hyperparameters. The search space explored during this process, including configurations for batch size, learning rates, activation functions, and more, is outlined in Table 2. This dynamic method effectively balances exploration (sampling across diverse possibilities) and exploitation (focusing on regions of high promise), leading to efficient optimization without being stuck in local optima. To further enhance efficiency, we integrated Hyperband Pruner, which terminates underperforming trials early and reallocates resources to the most promising configurations. This combination of random exploration, TPE refinement, and strategic pruning significantly improved the model’s performance. In practice, the TPE sampler concentrates trials in promising regions via density–ratio sampling and, when paired with the Hyperband Pruner, achieves faster, more sample-efficient convergence than grid/random search in our mixed continuous–discrete space. For further details on its implementation, please refer to the “Software Packages and Computational Tools” Section in the Supporting Information.

**Table 2:** Hyperparameter Search Space Explored for Model Optimization^a^,^b^

hyperparameter	search space
batch size	64, 128, 256
activation function	ReLU, ELU, Leaky ReLU, SELU, GELU, CELU, Swish, PReLU, ReLU6, RReLU
dropout rate (decoder)	0, 0.01, 0.1, 0.2
dropout rate (encoder)	0, 0.01, 0.1, 0.2
number of epochs (half cycle)	1, 2, 4
base learning rate	1e-3, 1e-4, 1e-5, 1e-6, 1e-7, 1e-8
max learning rate	0.5, 0.1, 0.05, 0.01, 0.005, 0.001, 0.0005, 0.0001, 0.00005
weight decay	1e-6, 5e-6, 1e-5, 5e-5, 1e-4, 5e-4, 1e-3, 5e-3, 1e-2, 5e-2
scheduler mode	Triangular, Triangular2
optimizer	AdamW, Adam, NAdam

a The table shows the defined ranges and options for critical hyperparameters, including architecture-specific configurations.

b These search spaces were designed to balance flexibility with domain relevance, ensuring a comprehensive exploration during optimization.

## 3 Results and Discussion

The pretrained model was evaluated on simulated spectra across eight CCD designs (see Figure S4 for more information.) To evaluate the robustness and generalizability of our model for etaloning correction, we performed a Leave-One-Design-Out Cross-Validation approach across eight different CCD designs. Data from one CCD design were held entirely out during training in each iteration and used solely for validation. This approach ensures that the model is tested on entirely unseen etaloning patterns, thereby providing a stringent evaluation of its generalization ability.

The results indicate that the model learns to correct etaloning patterns effectively in cases of interpolation, as seen in Designs 6–8, which involve variations in silicon thickness that fall within the range of the training data. However, the model struggles in cases of extrapolation, such as Designs 1–5, where new materials with different refractive indices introduce patterns not encountered during training. Notably, no hyperparameter tuning was performed during pretraining, as the primary goal was to use simulated data as a foundation for fine-tuning on real data. These observations highlight that while the model learns meaningful patterns, its ability to generalize entirely new patterns remains limited.

To demonstrate the impact of pretraining, we compared the TL model with a model trained only on real data. The availability of additional simulated data during pretraining provides the TL model with a more extensive data set, significantly enhancing its ability to generalize across various designs.

The gradient heatmaps in Figure 9 highlight the trends in correction efficacy. The TL model (Model 1) demonstrates significant improvements, particularly in interpolation scenarios (Designs 6–8), where SAM values improve by up to 68% and RMSE values reduce by 70–75%. These improvements indicate the model’s ability to reconstruct spectra for designs that are well represented in the training data accurately. In contrast, extrapolation scenarios such as Designs 1 and 4 show less pronounced improvements, with SAM and RMSE reductions in the 20–40% range, reflecting the challenges of correcting new etaloning patterns. For Design 8, the correction was most effective across all metrics, showcasing a 69% reduction in MAE and similar trends across RMSE and SAM, indicating minimal residual artifacts. On the other hand, Design 1 exhibited the least improvement, with MAE reducing by only 16% and SAM improvements remaining modest, reflecting the difficulty of handling completely unseen material properties.

**Figure 9:**
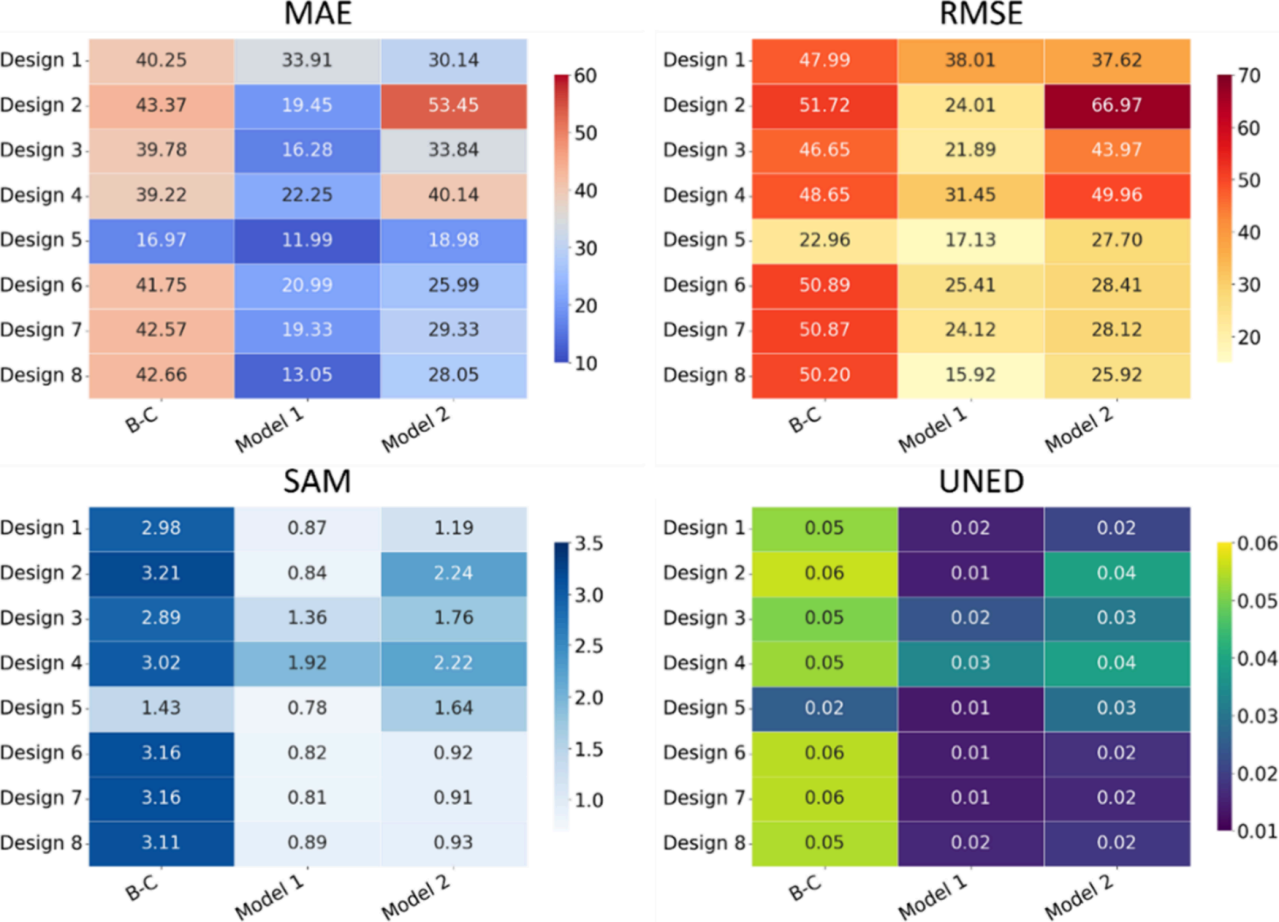
Gradient heatmaps for four metrics (SAM, RMSE, MAE, and UNED) derived from a Leave-One-Design-Out Cross-Validation. The heatmaps compare the transfer learning model (Model 1) and the real-data-only model (Model 2) across eight CCD designs. In interpolation scenarios (Designs 6–8), Model 1 shows up to 70% better performance than Model 2, while the difference is smaller in extrapolation cases (Designs 1–5).

The mean difference plots in Figure 10 provide more detailed insights into the correction quality, highlighting subtle variations that metrics such as SAM and RMSE did not reveal. While these metrics show that the real-data-only model can reduce errors compared to uncorrected data, the mean difference plots emphasize the strength of the transfer learning model in effectively minimizing etaloning artifacts and aligning closely with the true data. This difference is particularly evident in interpolation cases, such as Designs 6–8, where the transfer learning model consistently performs better. However, in extrapolation scenarios like Design 1, the mean difference plots expose the model’s limitations when faced with entirely new etaloning patterns. For completeness, we also include a baseline in the Supporting Information: Fixed window SG smoothing fails to remove etaloning without degrading peaks, whereas Model 1 performs best across the data set (see Figures S2 and S3).

**Figure 10:**
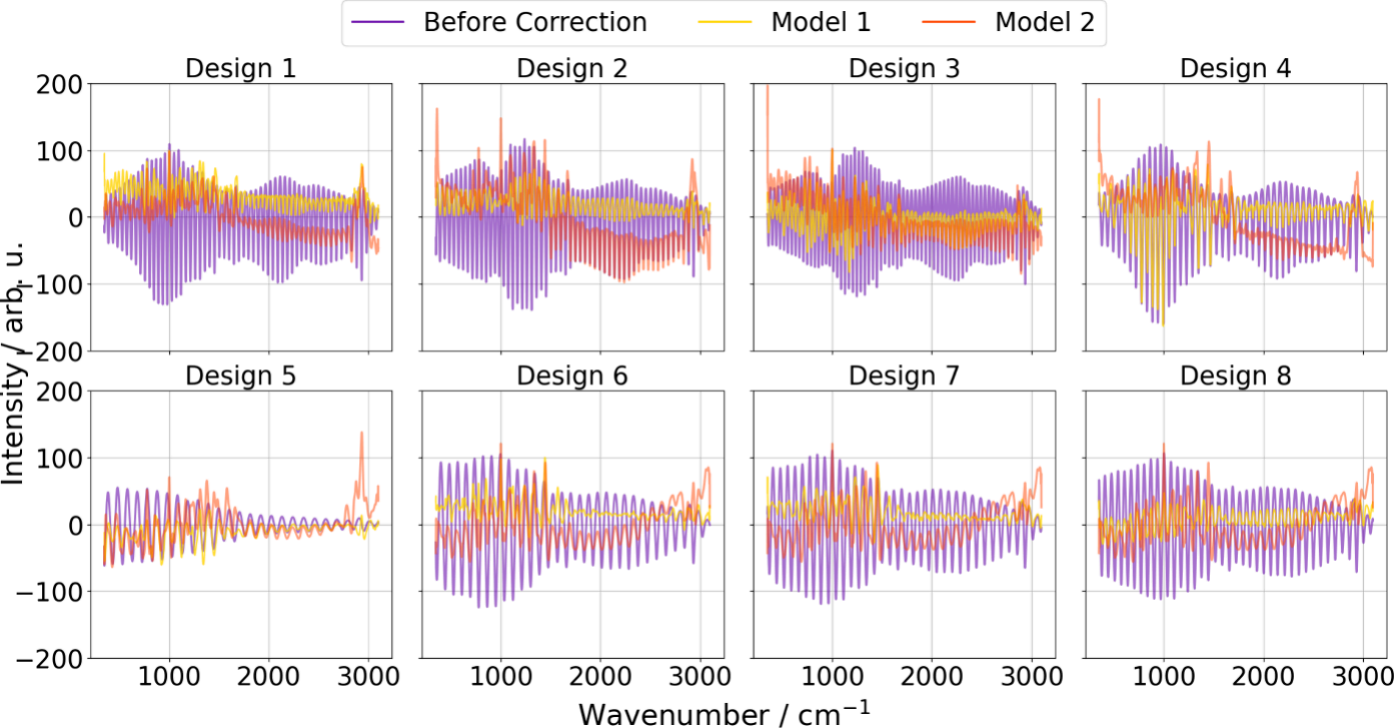
Spectral correction comparisons across eight CCD designs under a Leave-One-Design-Out Cross-Validation. Each panel presents the uncorrected spectra (purple), spectra corrected by Model 1 (yellow), and spectra corrected by Model 2 (orange). The figure illustrates that in interpolation cases (Designs 6–8), both models effectively reduce etaloning artifacts, with Model 1 yielding spectra closer to the ground truth, while Model 1 produces fewer residual artifacts in extrapolation scenarios.

Figure 11 provides further visual evidence of the TL model’s performance through individual spectra from Designs 4, 5, and 7. In interpolation cases such as Designs 5 and 7, the corrected spectra closely align with the ground truth with minimal deviations and smoother patterns. In Design 4, representing an extrapolation scenario, the corrected spectra exhibit more noticeable residual artifacts, aligning with the metric trends that indicate a less effective correction for designs involving new materials in CCD.

**Figure 11:**
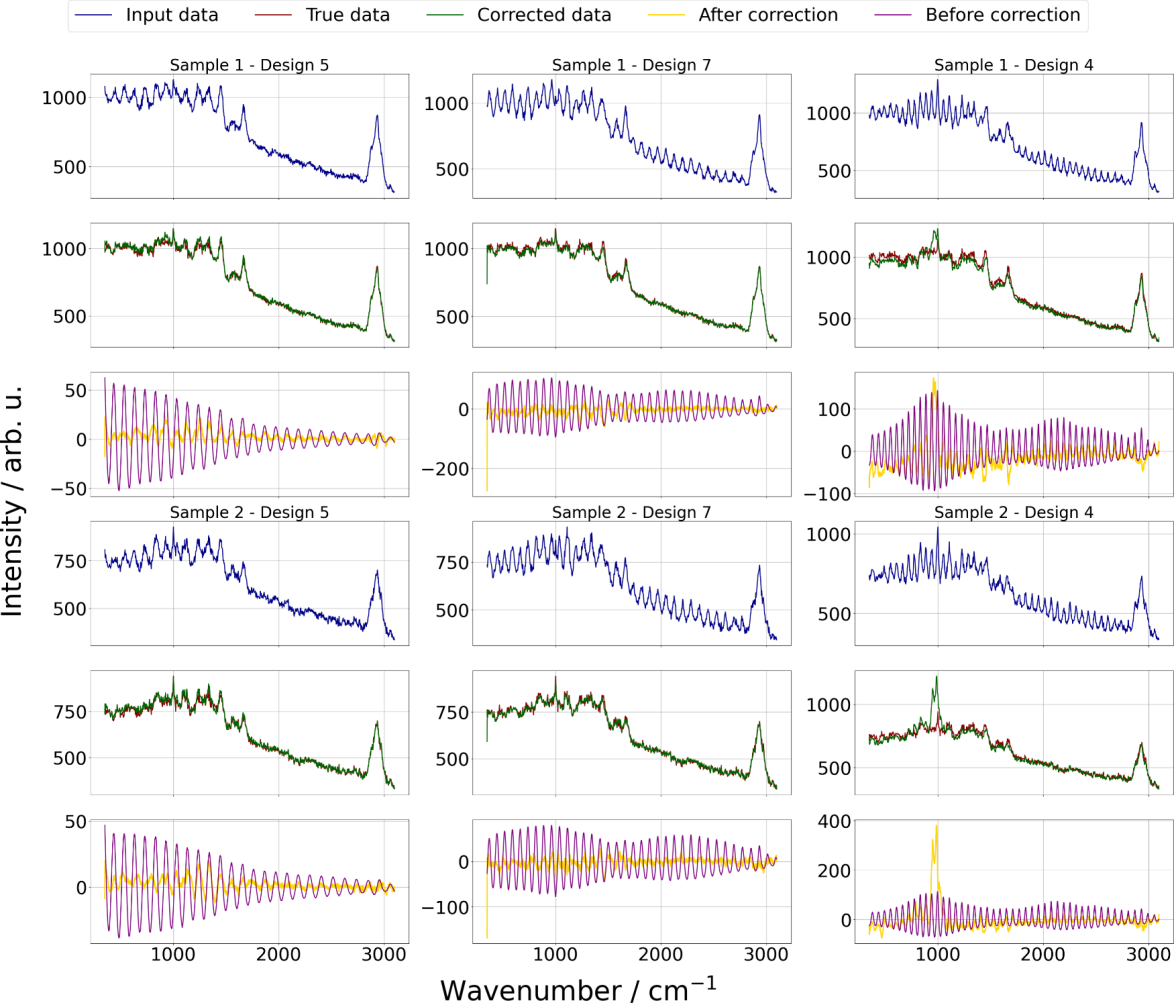
Comparison of spectral correction across three CCD designs (Designs 4, 5, and 7) for two random samples from the real data set. Each panel shows corrupted spectra (blue), true spectra (red), and corrected spectra (green). The difference between the corrected and true spectra (yellow) and the difference between the true and raw corrupted spectra (purple) are also shown. The plots highlight the effectiveness of the transfer learning model in aligning the corrected spectra with the true data. Notably, in the interpolation cases (Designs 5 and 7), the corrected spectra exhibit minimal deviations from the true spectra. The model captures the overall structure in extrapolation scenarios (e.g., Design 4), but residual artifacts remain, underscoring the challenges of addressing new etaloning patterns.

These findings underscore two critical insights: First, integrating simulated data during pretraining enhances the model’s capacity to learn and correct etaloning patterns, particularly in interpolation scenarios. Second, while quantitative metrics provide a valuable overview, qualitative analyses using mean difference plots offer essential insights into the practical efficacy of spectral correction, underscoring the superiority of the TL approach. Importantly, our observations reveal that the silicon layer thickness and material composition significantly influence model behavior. Designs with silicon thicknesses outside the pretraining range produce distinct interference features, while the presence of novel high-index materials (e.g., TiO_2_ and ZrO_2_) leads to new fringe patterns. Rather than indicating a shortcoming, these results highlight the model’s sensitivity to physically meaningful variations, reinforcing the importance of simulating diverse optical structures to ensure robust generalization.

## 4 Summary and Future Directions

This study demonstrates the strength of inverse modeling as a physics-respecting approach for correcting etaloning artifacts in Raman spectroscopy. By embedding the physical principles of etaloning directly into the learning process, inverse modeling provides interpretable and reliable corrections, overcoming the limitations of black box models. Integrated with forward modeling, which uses TMM simulations to generate realistic data sets, our dual-phase framework successfully addresses interpolation scenarios, particularly in CCD designs with silicon thickness variations. However, its limitations in extrapolation scenarios, such as complex designs involving new materials and complex layer combinations, underscore the need for further development.

Future work will focus on bridging this gap through targeted advancements. First, forward modeling will be expanded to simulate diverse etaloning patterns, including extreme cases and unexplored CCD layer configurations, while experimental data will be augmented with controlled perturbations to mimic unseen conditions. Additionally, we will explore the integration of multidimensional simulation approaches, such as finite-difference time-domain (FDTD) simulations, to address the limitations of one-dimensional models such as TMM and capture spatially resolved light–matter interactions within complex CCD geometries. Second, integrating PINNs will enhance generalization by embedding TMM simulations into the correction framework, ensuring precise artifact removal, even in challenging scenarios. Third, a continual learning approach will enable the model to adapt incrementally to new patterns while preserving previously acquired knowledge. Fourth, we will investigate cross-modal transfer learning and ensemble strategies, leveraging pretrained model weights to adapt to novel photonic modalities and improve generalization against data variability. These directions, aligned with our results, aim to establish robust artifact correction methods capable of handling diverse CCD designs, paving the way for more reliable and adaptable spectral analysis.

## Supplementary Material



## Data Availability

The inverse modeling framework utilized in this study, along with the pretrained model weights and the data set employed for training, is available in a GitHub repository at https://git.photonicdata.science/ravi_vulchi/inverse_modeling_etaloning.git. Additionally, the code for performing forward modeling is also included in the repository.

## Notes

Additional computational details for forward modeling (transfer matrix method implementation), inverse model training details (hyperparameter optimization with Optuna and computation environment), spectral data simulation protocol (GFN2-xTB workflow and Lorentzian line-broadening), baseline comparisons using Savitzky–Golay smoothing with parameter ranges, evaluation metrics definitions, Table S1 (CCD thin film designs with materials, layer stacks, and thicknesses), and Figures S1–S4 (SG smoothing examples, single-spectrum correction comparison, and data set-level RMSE analysis) (PDF)

## References

[1] Popp, J. ; Tuchin, V. V. ; Chiou, A. ; Heinemann, S. H. Photonics for health care; Wiley-VCH: 2012.

[2] Fini G. (2004). Applications
of Raman spectroscopy to pharmacy. J. Raman
Spectrosc..

[3] Cardona, M. ; Jusserand, B. Raman spectroscopy of vibrations in superlattices. In Light Scattering in Solids V: Superlattices and Other Microstructures; Springer: 2006, 49-152.

[4] Terry L. R., Sanders S., Potoff R. H., Kruel J. W., Jain M., Guo H. (2022). Applications of surface-enhanced Raman spectroscopy in environmental
detection. Analytical Science Advances.

[5] de
Oliveira Penido C. A. F., Pacheco M. T. T., Lednev I. K., Silveira L. (2016). Raman spectroscopy in forensic analysis: identification
of cocaine and other illegal drugs of abuse. J. Raman Spectrosc..

[6] Bowie, B. T. ; Chase, D. B. ; Lewis, I. R. ; Griffiths, P. R. Anomalies and artifacts in Raman Spectroscopy. In Handbook of vibrational spectroscopy; Wiley: 2002, 3.

[7] Hu B.-L., Zhang J., Cao K.-Q., Hao S.-J., Sun D.-X., Liu Y.-N. (2018). Research on the etalon effect in
dispersive hyperspectral
VNIR imagers using back-illuminated CCDs. IEEE
Transactions on Geoscience and Remote Sensing.

[8] Duponchel L., Bousquet B., Pelascini F., Motto-Ros V. (2020). Should we
prefer inverse models in quantitative LIBS analysis?. Journal of Analytical Atomic Spectrometry.

[9] Vogel, C. R. Computational methods for inverse problems; SIAM: 2002.

[10] Jiang A., Osamu Y., Chen L. (2020). Multilayer
optical thin film design
with deep Q learning. Sci. Rep..

[11] Luce A., Mahdavi A., Marquardt F., Wankerl H. (2022). TMM-Fast, a transfer
matrix computation package for multilayer thin-film optimization:
tutorial. JOSA A.

[12] Lou Q., Meng X., Karniadakis G. E. (2021). Physics-informed
neural networks
for solving forward and inverse flow problems via the Boltzmann-BGK
formulation. J. Comput. Phys..

[13] Kazemzadeh M., Martinez-Calderon M., Xu W., Chamley L. W., Hisey C. L., Broderick N. G. (2022). Cascaded
deep convolutional neural
networks as improved methods of preprocessing raman spectroscopy data. Anal. Chem..

[14] Zhuang F., Qi Z., Duan K., Xi D., Zhu Y., Zhu H., Xiong H., He Q. (2021). A comprehensive
survey on transfer
learning. Proceedings of the IEEE.

[15] Zardecki C., Dutta S., Goodsell D. S., Lowe R., Voigt M., Burley S. K. (2022). PDB-101: Educational
resources supporting
molecular explorations through biology and medicine. Protein Sci..

[16] Guo S., Bocklitz T., Neugebauer U., Popp J. (2017). Common mistakes in
cross-validating classification models. Analytical
Methods.

[17] Ali N., Girnus S., Rösch P., Popp J. r., Bocklitz T. (2018). Sample-size
planning for multivariate data: a Raman-spectroscopy-based example. Analytical chemistry.

[18] Rathmell C., Bingemann D., Zieg M., Creasey D. (2021). Portable Raman spectroscopy:
instrumentation and technology. Portable Spectroscopy
and Spectrometry.

[19] Heintz, R. Back illuminated vs. front illuminated CCD-based imaging sensors and how it impacts Raman spectra. Thermo Fisher Scientific (nd) 2019.

[20] Byrnes, S. J. Multilayer optical calculations. arXiv preprint arXiv:1603.02720 2016.

[21] Mohammed, Z. H. The Fresnel coefficient of thin film multilayer using transfer matrix method tmm. In IOP Conference Series: Materials Science and Engineering; IOP Publishing: 2019, Vol. 518, p032026.

[22] Groom D. E., Haque S., Holland S. E., Kolbe W. F. (2017). Quantum efficiency
modeling for a thick back-illuminated astronomical CCD. J. Appl. Phys..

