# Supplementary material for: Inverse Modeling for Artifact Removal in Photonic Data: A Computational Physics and Transfer Learning-Based Approach
Source: J Chem Inf Model. 2025 Oct 28;65(21):11733–45. doi: 10.1021/acs.jcim.5c02055 (PMC12606620; doi:10.1021/acs.jcim.5c02055)
Supplement: Supplementary file 1 [file ci5c02055_si_001.pdf]

Supporting Information: Inverse Modeling for Artifact Removal in Photonic Data: A Computational Physics and Transfer Learning-Based Approach

Ravi Teja Vulchi[1], Volodymyr Morgunov[1], Julian Hniopek[2], Oleg Ryabchykov[2], Thomas Bocklitz[1,2]

[1]Institute of Physical Chemistry (IPC) and Abbe Center of Photonics (ACP), Friedrich Schiller University Jena, Member of the Leibniz Centre for Photonics in Infection Research (LPI), Helmholtzweg 4, 07743 Jena, Germany.

[2]Leibniz Institute of Photonic Technology, Member of Leibniz Health Technologies, Member of the Leibniz Centre for Photonics in Infection Research (LPI), Albert-Einstein-Strasse 9, 07745 Jena, Germany.

Software Packages and Computational Tools

To model light propagation through layered optical structures, we utilize the Transfer Matrix Method (TMM), a well-established numerical method for analyzing electromagnetic wave behavior in stratified media comprising multiple thin layers [1]. The TMM enables the calculation of optical responses, such as reflection, transmission, and absorbance, over a wide range of wavelengths and angles of incidence, providing insights into the interaction of light with layered structures. We utilize the tmm_fast library, an advanced implementation of TMM designed for parallel computation of reflection and transmission for coherent light spectra that traverse multilayer thin films with dispersive materials. This package, built on PyTorch, leverages GPU acceleration, Autograd for gradient calculations, and vectorized operations using the Einstein summation convention, allowing us to handle the computational demands of evaluating light interactions with multilayer thin films. Depending on the number of layers and the considered wavelengths under specific angles of incidence, tmm_fast can reduce computation time by 2-3 orders of magnitude, a claim supported by cross-checks conducted with the code [2, 3]. We discuss the TMM implementation in detail in Section 2.3.2, and additional references for the tools used are provided in [2].

Inverse modeling was implemented using PyTorch, an open-source framework known for its scalability and flexibility in developing machine learning architectures. Hyperparameter optimization was systematically conducted using Optuna, a robust framework that enabled efficient fine-tuning of the DL model's parameters, further enhancing its performance [4]. The DL model's training and hyperparameter tuning were conducted on GPU nodes equipped with NVIDIA A100 GPUs, ensuring efficient handling of computational tasks.

Spectral Data Simulation

As all experimental spectrum shows changes compared to a theoretical pure component spectrum due to interactions with the optical environment (e.g., diffraction), simulated data was used to isolate the effects of artifacts modeled in this study.

Spectral data was generated using the GFN2-xTB semi-empirical quantum mechanical method. As a semi-empirical method, it employs the same ab initio framework as the Hartree–Fock method. However, it replaces some complex integrals with empirical parameterizations to reduce computational cost. In GFN2-xTB specifically, the correlation integrals are approximated using a tight-binding scheme, removing the most computationally intensive steps. Empirical corrections for dispersion and electrostatic interactions are then applied to retain reasonable accuracy. This balance between computational efficiency and accuracy allows large-scale spectral calculations on standard hardware, enabling coverage of the chemical diversity required for inverse model training.

We used the xtb package (v6.5.1) to generate the data developed by the Grimme Group [5]. Each molecule from the World Wide Protein Data Bank small-molecule set was first geometry optimized starting from real-world coordinates,

using tight convergence criteria (E_{conv}/E_h = $5{\cdot}10^{-6}$, $G_{conv}/(E_h\, \alpha\,)$= $2{\cdot}10^{-4}$). Vibrational frequencies were then computed using the ohess keyword. Molecules with imaginary modes were excluded from the dataset. The final line spectra were broadened using Lorentzian functions with randomly sampled full width at half maximum values to simulate experimental line shapes due to natural, Doppler, and pressure broadening.

Savitzky–Golay Smoothing as a Baseline for Etaloning Removal

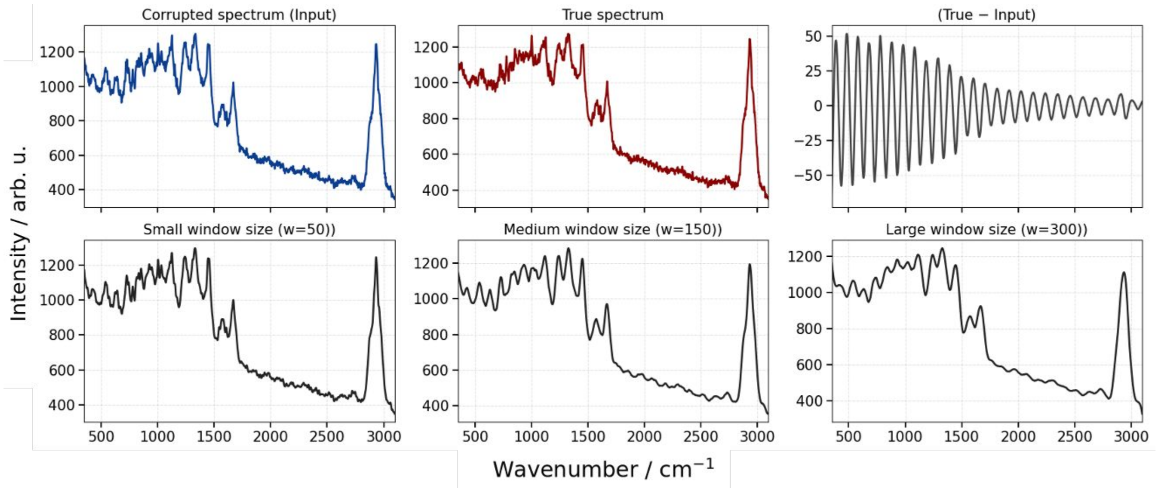

Figure S1. Example output of Savitzky–Golay smoothing applied to one Raman spectrum affected by etaloning. Top row: Corrupted spectra with etaloning (blue), True spectra (red), and the difference between them. Bottom row: SG-smoothed outputs with increasing window sizes. Narrow windows preserve detail but fail to remove interference; larger windows suppress the fringes but distort peak shapes and band intensities.

We use Savitzky–Golay (SG) smoothing as a simple, non-physics-based method to remove etaloning from Raman spectra (Figure S1). With a small SG window size, the fringes remain clearly visible across the fingerprint and high-wavenumber regions because the window size is too small to suppress the interference. With a medium window size, the fringes are reduced, but narrow features start to blur, and small peaks lose contrast. Using a large window removes most of the interference, but it also eliminates fine spectral structure, flattening vibrational peaks and distorting chemical information.

These outcomes reflect a fundamental limitation: there is no single SG window that can simultaneously remove interference and preserve narrow Raman peaks. Because these fringes can overlap and change across wavenumbers, any fixed window size either leaves the fringes visible or removes the peaks. In practice, SG would require tuning the window for each spectrum, which makes it impractical for real-world workflows. These limitations are also evident when compared to the results from our DL model. Figure S2 illustrates this contrast; small peaks are often most affected by SG smoothing. The DL-corrected output closely matches the true Raman spectrum across the full wavenumber range. The DL model effectively removes etaloning artifacts without compromising vibrational peak shapes, thereby preserving both peak positions and relative intensities.

To evaluate performance at the dataset level, we calculated the mean RMSE across all spectra (Figure S3). SG filtering with three different windows, where each of these values is higher than that of the uncorrected spectra. In contrast, DL-corrected spectra achieve a significantly lower RMSE of 17.13, indicating that our model removes fringe patterns more effectively while retaining meaningful chemical information. These results demonstrate the clear advantage of using a physics-informed deep learning model over fixed-window filtering for automated etaloning correction in Raman spectroscopy.

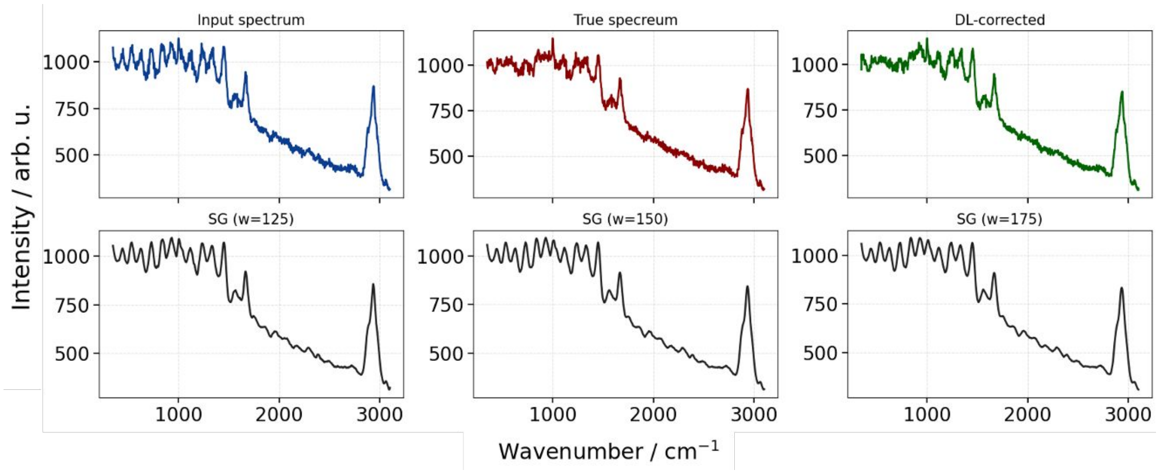

Figure S2. Single-spectrum comparison of etaloning correction. The top row shows the input spectrum with etaloning (blue), the ground truth (red), and the DL-corrected output (green). Bottom row shows SG results with window sizes of 125, 150, and 175. SG smoothing cannot remove etaloning without degrading spectral detail, and the DL-corrected output closely follows the true spectrum.

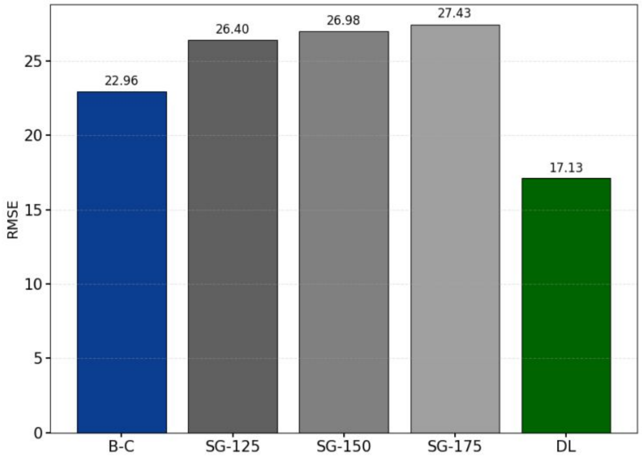

Figure S3. Comparison of average RMSE values for corrupted spectra before correction (B-C), SG filtering at three window sizes, and the DL-corrected output. All SG window sizes result in higher errors than the uncorrected input. The DL model achieves the lowest RMSE, reflecting its ability to remove fringe patterns while preserving spectral details.

Transfer Matrix Method as the Forward Modeling Framework

The selection of the TMM module for forward modeling was driven by its unique combination of physical accuracy, computational efficiency, and versatility in simulating multilayered optical systems. Several characteristics of TMM closely align with the specific modeling challenges addressed in this study [1, 6]:

- **Efficient Modeling of Multilayer Systems:** TMM enables fast computation of reflection, transmission, and absorption co-efficients across multilayers with arbitrary ordering, accommodating the complexity of modern thin-film architectures.
- **Accurate Treatment of Complex Refractive Indices:** The method effectively incorporates materials with complex refractive indices, enabling accurate modeling of absorption phenomena. In the special case of purely real refractive indices, TMM faithfully represents energy-conserving, lossless behavior.
- **Adaptability to Varying Layer Thicknesses:** TMM supports layers of any thickness, from nanometers to microns, allowing it to capture both coherent and partially incoherent optical effects as required by the system.

- **Electric Field Profiling Across Structures:** Beyond spectral calculations, TMM also calculates the spatial distribution of electric fields within multilayer stacks, providing deeper insights into light–matter interactions at interfaces.
- **Simplified Boundary Conditions Through Semi-Infinite Layers:** The method assumes that each layer extends infinitely in the lateral directions and treats the incident and substrate media as semi-infinite, simplifying boundary condition management without sacrificing generality.
- **Linear Scaling of Computational Cost:** TMM propagates fields sequentially through matrix multiplication across each layer, resulting in computational costs that scale linearly with the number of layers. This property balances simulation speed and accuracy for complex multilayer systems.
- **Support for Dispersive and Periodic Systems:** TMM effectively handles dispersion relations for crystals and periodic multilayer systems, extending its applicability to various optical materials.

Comparative analyses against other modeling approaches have further confirmed the advantages of TMM, especially regarding accuracy, computational efficiency, and versatility for thin-film optical simulations [7]. However, several inherent limitations should be acknowledged, including its one-dimensional approximation, assumptions of uniform layer thicknesses and ideal interfaces, neglect of surface roughness, scattering phenomena, and deviations in material properties. These simplifications might lead to discrepancies between simulated results and real-world experimental observations, especially in highly sensitive scenarios. Despite these limitations, TMM remains a highly suitable and effective framework for addressing the specific forward modeling requirements in this study. Future work may consider enhancing its accuracy by integrating multidimensional modeling techniques or empirical characterization methods.

Spectral Markers and Evaluation Metrics

The etaloning effect is a wavelength-dependent interference phenomenon that introduces periodic oscillations in spectral intensities, scaling with the baseline intensity. While the etaloning does not alter the peak positions in the spectra, it distorts the shapes of the peaks as the interference modifies intensity at specific wavelengths. To rigorously evaluate the effectiveness of the correction procedure, it is imperative to utilize spectral markers that extend beyond traditional intensity-based metrics such as Root Mean Square Error (RMSE) and Mean Absolute Error (MAE). These conventional metrics provide a quantitative measure of absolute intensity deviations between corrected and true spectra, with RMSE highlighting more significant variations and MAE offering a general measure of average error:

$$\text{RMSE} = \sqrt{\frac{1}{n}\sum_{i=1}^{n}\left(T_i - C_i\right)^2},\ \text{MAE} = \frac{1}{n}\sum_{i=1}^{n}|T_i - C_i|$$

T_i and C_i are the true and corrected spectra intensities at the i_{th} wavenumber. However, etaloning distorts peak shapes by introducing periodic oscillations in spectral intensities without shifting peak positions. Metrics like RMSE and MAE, while valuable for quantifying overall intensity errors, fail to capture the nuanced recovery of spectral shapes and proportional relationships, which are critical to the spectral analysis.

We employ the Spectral Angle Mapper (SAM), a shape-sensitive metric that calculates the angular difference between the true and corrected spectra treated as vectors to address this limitation. SAM is invariant to baseline variations and intensity scaling, making it ideal for evaluating proportional relationships in spectral shapes.[8].Commonly used in hyperspectral analysis, SAM robustly assesses how well the corrected spectra restore true patterns distorted by etaloning. Complementing SAM, the Unit Normalized Euclidean Distance (UNED) quantifies residual deviations between the normalized true and corrected spectra:

$$\theta = \cos^{-1}\left(\frac{\sum_{i=1}^{n} T_i C_i}{\sqrt{\sum_{i=1}^{n} T_i^2}\cdot\sqrt{\sum_{i=1}^{n} C_i^2}}\right),\quad \text{UNED} = \sqrt{\sum_{i=1}^{n}\left(\frac{T_i}{|T|} - \frac{C_i}{|C|}\right)^2}$$

Where $|T| = \sqrt{\sum_{i=1}^{n} T_i^2}$,$|C| = \sqrt{\sum_{i=1}^{n} C_i^2}$ are the norms of the true and corrected spectra, respectively. By normalizing both spectra, UNED isolates the oscillatory distortions from baseline effects, effectively measuring artifact reduction.

SAM and UNED provide complementary evaluations. SAM validates the recovery of proportional spectral shapes, while UNED quantifies the magnitude of periodic distortions removed [9]. This dual approach ensures the correction method restores both global alignment and localized spectral features, focusing exclusively on removing etaloning artifacts without addressing inherent baseline variability.

Tables

Tables S1. Material properties of CCD designs that illustrate interpolation and extrapolation scenarios. These designs exemplify the structural variations and material compositions employed in cross-validation to assess the model's capacity to manage familiar and unfamiliar etaloning patterns.

**Designs**	**Number of layers**	**Materials used**	**Thickness of layers (in order)**
Design 1	8	ITO, SiO2, ISDP, SiO_2 (additional), Si_3N_4, Polysilicon gates, Additional SiO_2, Silicon substrate	58 nm, 80 nm, 25 nm, 50 nm, 50 nm, 300 nm, 2 µm, 250 µm
Design 2	9	ITO, ZrO_2, SiO2, ISDP, SiO_2 (additional), Si_3N_4, Polysilicon gates, Additional SiO_2, Silicon substrate	20 nm, 38 nm, 106 nm, 11 nm, 50 nm, 50 nm, 300 nm, 2 µm, 250 µm
Design 3	9	ITO, ZrO_2, SiO2, ISDP, SiO_2 (additional), Si_3N_4, Polysilicon gates, Additional SiO_2, Silicon substrate	20 nm, 38 nm, 118 nm, 18 nm, 50 nm, 50 nm, 300 nm, 2 µm, 320 µm
Design 5	5	ITO, ZrO_2, SiO_2, ISDP, Silicon substrate	15 nm, 35 nm, 70 nm, 10 µm, 100 nm
Design 6	7	ITO, SiO_2, ISDP, Silicon bulk, SiO_2, Si_3N_4, Silicon substrate	80 nm, 55 nm, 10 nm, 14 µm, 80 nm, 240 nm, 500 nm
Design 8	7	ITO, SiO_2, ISDP, Silicon bulk, SiO_2, Si_3N_4, Silicon substrate	80 nm, 55 nm, 10 nm, 18 µm, 80 nm, 240 nm, 500 nm

Figures

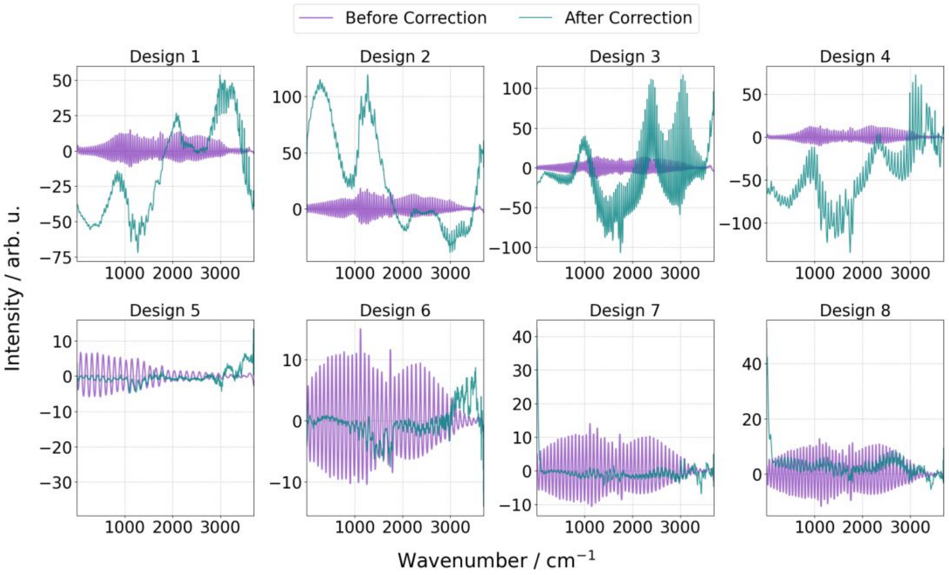

Figure S4. Pre-trained model performance across eight CCD designs, comparing spectra before and after correction. Designs 6, 7, and 8, which involve variations in silicon thickness, exhibit successful etaloning correction as the corrected spectra align closely with the baseline. In contrast, Designs 1 to 5, which include new materials with different refractive indices, show residual etaloning patterns after correction, showing limited extrapolation capability. These results demonstrate the model's ability to interpolate within known patterns and highlight its limitations in generalizing to novel scenarios. Each subplot corresponds to one CCD design, emphasizing the strengths and weaknesses of the pre-trained model's correction ability.

References

(1) Katsidis, C. C.; Siapkas, D. I. General transfer-matrix method for optical multilayer systems with coherent, partially coherent, and incoherent interference. *Applied optics* **2002**, *41* (19), 3978-3987.

(2) Luce, A.; Mahdavi, A.; Marquardt, F.; Wankerl, H. TMM-Fast, a transfer matrix computation package for multilayer thin-film optimization: tutorial. *JOSA A* **2022**, *39* (6), 1007-1013.

(3) Byrnes, S. J. Multilayer optical calculations. *arXiv preprint arXiv:1603.02720* **2016**.

(4) Akiba, T.; Sano, S.; Yanase, T.; Ohta, T.; Koyama, M. Optuna: A next-generation hyperparameter optimization framework. In *Proceedings of the 25th ACM SIGKDD international conference on knowledge discovery & data mining*, 2019; pp 2623-2631.

(5) Bannwarth, C.; Ehlert, S.; Grimme, S. GFN2-xTB—An accurate and broadly parametrized self-consistent tight-binding quantum chemical method with multipole electrostatics and density-dependent dispersion contributions. *Journal of chemical theory and computation* **2019**, *15* (3), 1652-1671.

(6) Acquaroli, L. N. Matrix method for thin film optics. *arXiv preprint arXiv:1809.07708* **2018**.

(7) Jo, J.; Jung, E.; Park, J. C.; Hwang, J. Comparative study of optical analysis methods for thin films. *Current Applied Physics* **2020**, *20* (2), 237-243.

(8) Dennison, P. E.; Halligan, K. Q.; Roberts, D. A. A comparison of error metrics and constraints for multiple endmember spectral mixture analysis and spectral angle mapper. *Remote Sensing of Environment* **2004**, *93* (3), 359-367.
(9) Samuel, A. Z.; Mukojima, R.; Horii, S.; Ando, M.; Egashira, S.; Nakashima, T.; Iwatsuki, M.; Takeyama, H. On selecting a suitable spectral matching method for automated analytical applications of Raman spectroscopy. *ACS omega* **2021**, *6* (3), 2060-2065.
